# Optokinetic stimulation induces vertical vergence, possibly through a non-visual pathway

**DOI:** 10.1038/s41598-020-72646-8

**Published:** 2020-09-23

**Authors:** Tobias Wibble, Tony Pansell

**Affiliations:** 1 Department of Clinical Neuroscience, Division of Eye and Vision, Marianne Bernadotte Centre, Karolinska Institutet, Stockholm, Sweden; 2 St Erik Eye Hospital, Stockholm, Sweden

**Keywords:** Oculomotor system, Visual system

## Abstract

Vertical vergence is generally associated with one of three mechanisms: vestibular activation during a head tilt, induced by vertical visual disparity, or as a by-product of ocular torsion. However, vertical vergence can also be induced by seemingly unrelated visual conditions, such as optokinetic rotations. This study aims to investigate the effect of vision on this latter form of vertical vergence. Eight subjects (4m/4f) viewed a visual scene in head erect position in two different viewing conditions (monocular and binocular). The scene, containing white lines angled at 45° against a black background, was projected at an eye-screen distance of 2 m, and rotated 28° at an acceleration of 56°/s^2^. Eye movements were recorded using a Chronos Eye-Tracker, and eye occlusions were carried out by placing an infrared-translucent cover in front of the left eye during monocular viewing. Results revealed vergence amplitudes during binocular viewing to be significantly lower than those seen for monocular conditions (*p* = 0.003), while torsion remained unaffected. This indicates that vertical vergence to optokinetic stimulation, though visually induced, is visually suppressed during binocular viewing. Considering that vertical vergence is generally viewed as a vestibular signal, the findings may reflect a visually induced activation of a vestibular pathway.

## Introduction

Single binocular vision depends on the brain’s capacity to efficiently adjust the relative position of right and left eye visual input by vergence eye movements (motor fusion), and to cortically fuse the two images into one single binocular percept (sensory fusion). When vergence eye movements are unable to adjust the eye position adequately or sensory fusion is abolished, diplopia occurs. During visual fixation of a stationary target, the eyes will be moved into position by vergence eye movements, which will then be maintained by tonic muscle activity. As the viewed target moves in-depth, our eyes will adjust, altering the level of torsional, horizontal, and vertical vergence as to maintain binocularity.

Physiologically, a vertical vergence is most often associated with a vestibular signal indicating a head tilt, with the contralateral eye being depressed and the ipsilateral elevated^1^. The extent of this skewing response is dependent on the subject’s fusional range, as its purpose is to avoid the breakdown of binocularity^2^. However, we have previously shown that a rotating visual image, simulating the optokinetic field-of-view as seen during a head rotation, will also lead to a vertical vergence^3,4^. This eye movement is also accompanied by ocular torsion in the same direction as the visual rotation^5^. While this torsional response seems insensitive to the acceleration of the visual rotation, vergence has proven comparably accommodating in showing decreased vergence to increased accelerations^6^.

Vertical eye movements exhibit a close relationship with ocular torsion, which can be expected due to the mechanical constraints of the extraocular muscles. This relationship has been well-described, particularly by Schor, whose extensive use of prisms has provided valuable insight into the orbital dynamics allowing for the coupling of torsional and vergence eye movements^7,8^. In addition to the vestibular response seen during head tilts, vertical visual disparities will induce a combination of torsion and vertical vergence, which has been associated with less reliable depth-perception^9,10^.

Despite the intrinsic physiological link between torsion and vertical vergence, evidence generally points to different neural pathways of induction during optokinetic roll stimulations, with torsion showing a greater dependency on visual information density^3,11^. This is also true from a vestibular perspective, with the otolith organs sensing static head position and inducing vertical vergence, while dynamic head movements are relayed through the semi-circular canals, which lead to ocular counter-rolling^12,13^. Additionally, prolonged monocular occlusion aiming to position each eye at a natural resting state has shown that the direction of vertical and torsional phorias are independent of one another^14^.

Visually induced torsional vergence can be readily observed during binocular conditions when viewing dichoptic rotating scenes, exhibiting a gain of 0.2 during low-frequency oscillations at low amplitudes^15^. In a monocular setting, when the visual drive of binocular fusion has been eliminated, this response shows an increased variability^16^. As vertical vergence also serves to fuse the visual fields, one may expect a similar response pattern, albeit of different ratios. There also exists some evidence of independent visual factors influencing vertical vergence: In a setting involving subjects performing rapid eye movements between two vertically aligned targets, one of which was closer to one of the eyes, one can expect a vergence response due to the visual disparity. However, this response is retained even if this disparity is removed with the aid of prisms^17^. As vertical vergence to optokinetic rotations has only recently been shown, there are currently few studies outlining the conditions during which it can be observed.

This study aims to explore the influence of vision on vertical vergence to optokinetic rotations through evaluating the effect of binocular and monocular viewing conditions. If the observed vergence is purposed to increase the vertical fusion range, we hypothesise that occlusion of one eye will reduce or abolish the response. Should the divergence response be unaffected by the viewing condition, it may instead indicate that the eye movement is purely secondary to the torsional response. If the divergence response increases to occlusion, we put forward that it is driven by vestibular activation and dampened by visual vergence control. The findings will be compared to the torsional response, and discussed in the context of expected neural mechanisms.

## Material and methods

### Subjects

Eight healthy subjects were recruited for this study (4 male, 4 female), having no history of balance disorders, taking no medication affecting the central nervous system, normal eye motility, corrected visual acuity (VA ≥ 1.0), no latent vertical deviations or phorias (no vertical deviation during cover test), and stereoscopic vision equal to or better than 60″ (TNO). The research was carried out in accordance with the Declaration of Helsinki. Informed consent was obtained from all subjects after explanation of the nature and possible consequences of the study. The research was approved by the Regional Ethics Committee of Stockholm (EPN 2018-1768-31-1).

### Method

The test procedure consisted of subjects viewing a rotating visual scene standing at an eye-screen distance of 2 m under both monocular and binocular viewing conditions. Subjects were presented to both clockwise (CW) and counter-clockwise (CCW) rotations of the visual scene around a central fixation pivot point. Each subject was exposed to the four test conditions once, the order of which was balanced between subjects though stratified randomization; half the participants started with the CW direction, half of which started with the monocular viewing condition and vice versa. All monocular viewing conditions were performed with the left eye covered.

**Figure 1:**
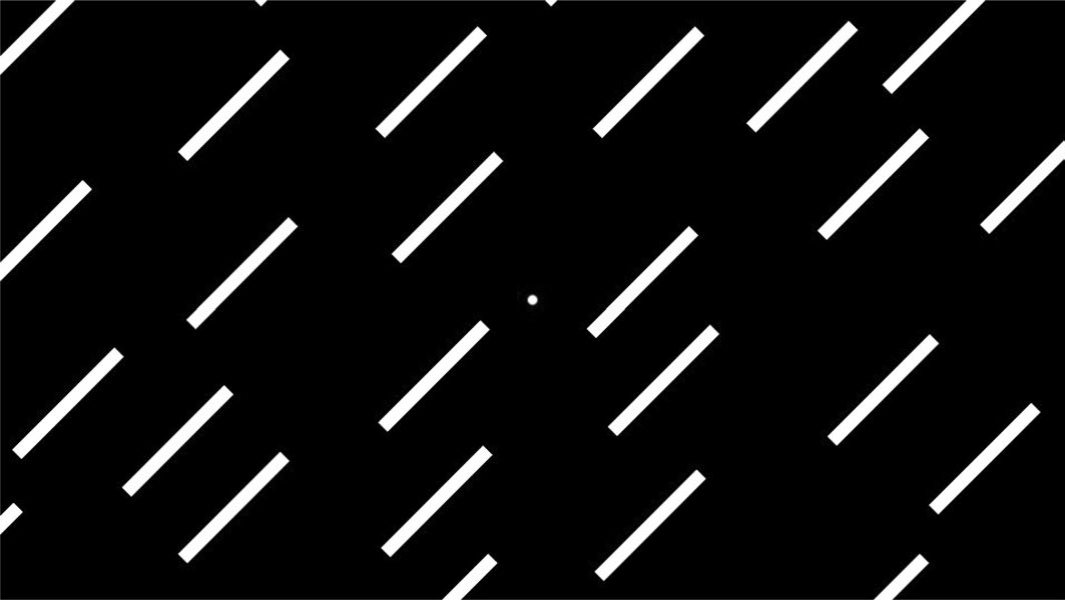
The visual stimulation covered 50° of subjects’ field of vision, and consisted of 38 white lines, 0.42 cm wide and 3.25 cm long (visual angle, 0.93°) standing at an angle of 45°, rotating around a central fixation point of 0.32 cm in diameter. The image rotated 28° during the active stimulation phase.

The visual stimulation consisted of white lines against a black background, presenting the lines at a tilted angle (Fig. 1), which has been previously shown to produce distinct vertical vergence of the eyes when rotated^3^. The rotation amplitude was set to 28° with an acceleration of 56°/s^2^. Each trial started with the subject viewing the static image (see Fig. 1) for 20 s, after which the motion was initiated. The visual scene was then kept static at its final position for another 20 s. These parameters have been shown to produce reliable eye-movement responses during in-lab trials.

Eye movements were recorded using the head mounted Chronos Eye Tracker Device (C-ETD; Chronos Inc, Berlin, Germany), performing binocular recordings at 100 Hz with a spatial resolution for vertical eye movements of < 0.05° and torsional eye movements of < 0.1°. Horizontal and vertical eye movements were calculated by means of pupil displacement, calibrated to each test person by initially having them perform an eye movement pattern to a series of dots with known separations, allowing for translating video captured pupil displacement into degrees of eye rotation. This video-based eye tracking has been previously established for this type of visual stimulation^3^. The head mount also carries a head tracking system, allowing for recording head accelerations in six dimensions (three rotational and three translational).

Eye movement analysis was carried out through subtracting the position of the right pupil from the position of the left. This yielded the vertical alignment of the eyes, with any value deviating from zero signifying a vertical vergence in either direction, with a positive value indicating left-over-right. As torsional eye movements are generally associated with vertical vergence, ocular torsion was also calculated in order to analyse the correlation between them. Torsional eye movements were analysed through measuring iris displacement around the centre of the pupil as tracked by the Chronos Eye Tracker. The quality of each torsional frame was automatically indexed to a value between 0 and 1, with 1 being the highest. This data was carried over to Origin (OriginPro 2017, OriginLab, Northampton, MA), and plotted against time, and torsional frames below a quality of 0.5 were removed to ascertain a good signal quality. Consequently, the amplitudes could be calculated from baseline, i.e. prior to stimulus rotation. The peak amplitude was recorded as the highest point of the eye movement slow-phase during the active rotation of the visual scene. Binocular recordings of the eyes during monocular viewing was carried out by fixing an infrared-translucent cover sheath to the mask in front of subjects’ left eyes. This effectively blocked visual input on that eye as the sheath would appear black to the subject, while simultaneously allowing the infrared camera of the video-tracker to record through the material.

## Results

Paired T-tests revealed no significant differences between CCW and CW stimulus rotation for neither vertical vergence (t(7) = 0.079; *p* = 0.93) nor ocular torsion (t(7) = 1.176; *p* = 0.278), allowing for further analysis to be indiscriminative of rotation direction. Similarly, there were no significant effect of testing order for vertical vergence (t(7) = 0.225; *p* = 0.827) or ocular torsion (t(7) = 0.213; *p* = 0.837).

**Figure 2:**
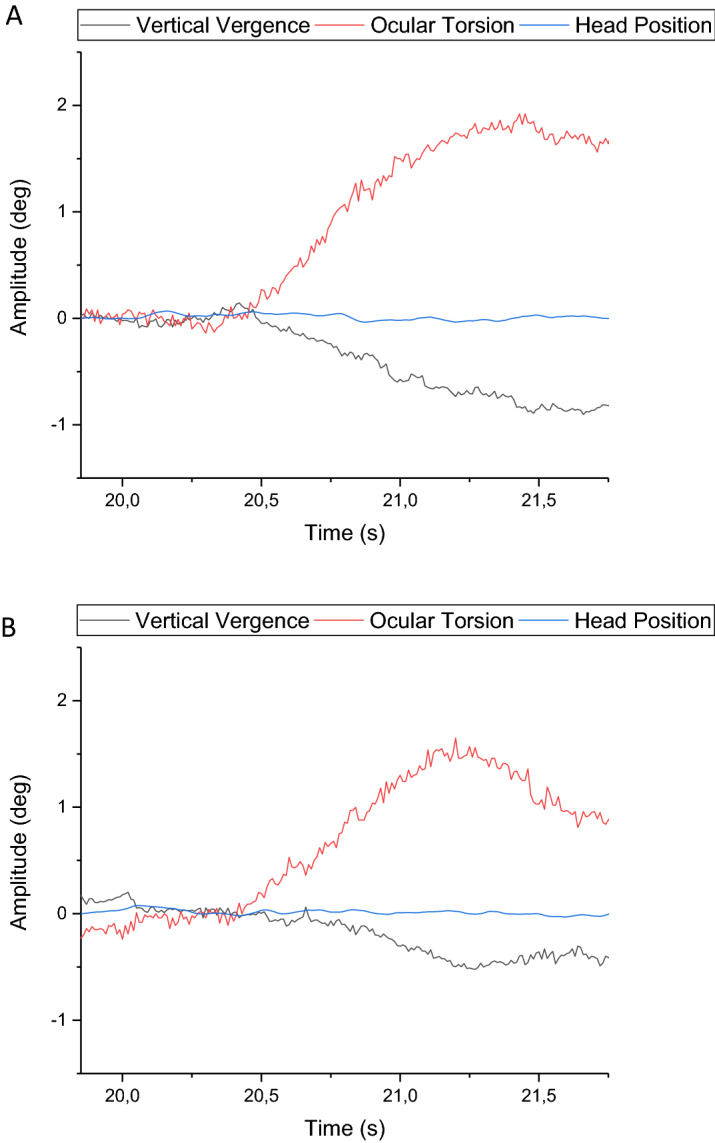
The unaltered eye movement response as well as head position during monocular viewing conditions (**A**) and binocular viewing conditions (**B**) for the counter clockwise rotation direction, as seen for subject 4.

Vertical vergence amplitudes (see Table 1) were normally distributed for both binocular and monocular viewing conditions. A paired-samples T-test revealed a significant difference between binocular and monocular viewing conditions (t(7) = 4.58; *p* = 0.003). An example of this increased amplitude during monocular viewing can be seen in Fig. 2. Additionally, a repeated multivariate analysis revealed a significant within-subject effect of viewing condition (*F* (1, 7) = 20.678; *p* = 0.003).

**Table 1:** The signal amplitude seen to binocular and monocular viewing conditions for both vertical vergence and torsional eye movements.

Subject	Divergence binocular	Divergence monocular	Torsion binocular	Torsion monocular
1	0.36	0.73	0.47	0.79
2	0.35	0.67	0.37	0.80
3	0.24	0.54	1.08	0.89
4	0.42	0.66	1.62	0.94
5	0.58	0.55	0.98	0.90
6	0.72	0.77	1.28	0.55
7	0.42	0.74	0.55	2.07
8	0.51	0.83	0.19	0.86
Mean	0.45	0.69	0.81	0.97
SD	0.15	0.10	0.50	0.46

Values are given as amplitudes in degrees, as seen during the rotation of the visual scene. Each column represent average values between CCW and CW directions for each subject.

The torsional response exhibited no significant differences between monocular and binocular viewing conditions (t(7) = 0.597; *p* = 0.567). The mean torsional response was 0.97° (SD 0.65) for monocular and 0.81° (SD 0.54) for binocular viewing conditions.

A correlation analysis between vertical vergence and ocular torsion did not reveal any strong relation for neither monocular (r^2^ = 0.05) nor binocular (r^2^ = 0.009) viewing conditions. When analysing the correlation of the differences between viewing conditions (monocular vergence − binocular vergence correlated to monocular torsion − binocular torsion), a positive correlation (r^2^ = 0.30) was found, indicating that a larger torsional response seen in monocular viewing was associated with a larger vertical vergence response in monocular viewing. When subject to within-subject analysis, there was a significant difference between the torsional and vergence responses (*F* (1, 7) = 7.706; *p* = 0.027), indicating separate response patterns between the two eye movements.

## Discussion

The aim of this study was to provide a basic description of how visually induced vertical vergence is affected by binocularity. Results support the hypothesis that binocular fusion reduces the vertical vergence, as the vergence response was significantly increased during monocular viewing conditions despite ocular torsion remaining the same between conditions.

Binocularity necessitates a strict control of vergence eye movements for foveated animals. As the purpose of vertical vergence is to increase the fusional range during a head rotation, it can consequently be expected that this control would be hampered when the risk of diplopia is removed by occluding one eye. Still, as the visual stimulus used in this study presented a stable, unmoving, fixation point and no vertical disparity, it is not completely clear why there would be any vertical vergence at all. One may surmise that the primary reason for the vergence is to adjust the eyes in accordance to the shifting horizon during the visual rotation. However, during monocularity only one eye receives the information, meaning that no eye movement would be expected as the eyes cannot fuse. Ocular torsion, on the other hand, has a more direct relationship with the stimulation. As each retina will be subject to the rotation one may expect the eye movement to be less affected by the viewing condition, which is also represented in the findings of this study.

While the population recruited for this study was relatively few in numbers, results were highly consistent across subjects. Subjects were recruited to fill the need to balance the different trials, after which no further experiments were performed due to the onset of the covid-pandemic. As such, when expanding on this topic in future studies, it would be beneficial to increase the number of subjects so as to ensure greater power and reproducibility. Still, as response patterns were generally robust, the findings add important context for this poorly understood mechanism.

A possible explanation for the vertical vergence could be its intrinsic link to the torsional eye movement. As previously noted, due the orbital mechanics of the extraocular muscles, there can be neither torsion nor vertical vergence without the other. This phenomenon was well-described already in the seventies, when studies involving head tilts, forced cyclofusion through prisms, as well as having subjects monocularly view a rotating visual scene all produced secondary vertical vergence of the eyes^18^. While the amplitude of the latter rotations was 60°, it produced an average vertical vergence of 1.1 prism dioptres (0.63°). This roughly corresponds to the results of the monocular viewing conditions in this study, despite the amplitude being more than double^19^. As indicated by the correlation analysis carried out in the present study, the mechanical link between the two eye movements can only account for a portion of the findings, approximately 30%.

Additionally, the within-subject analysis indicated that vergence and torsional eye movements behave significantly different from one another. It is therefore clear that vertical vergence is not necessarily only seen as a by-product of torsion during optokinetic rotations, even in the absence of visual disparity. Still, the torsion-vergence relationship is not altogether clear, and it is also important to take note that vertical vergence caused by visual disparities cause secondary torsion, as the oblique muscles are the primary contributors to fusional vertical vergence^20^. Naturally, the purpose of the eye movement is highly relevant to what eye movement can be considered secondary. It is worth mentioning that even during the viewing of dichoptic stimulations forcing a fusional vergence, the eye movements are spatiotemporally unsymmetrical according to Hering’s Law of Equal Innervation^21^, further highlighting the complex nature of orbital mechanics and its multisensory influences.

Considering the different response patterns of vertical vergence and torsion, it would seem likely that the two eye movements have different integrational pathways. As stated in the introduction, the causes for vertical vergence is generally attributed to either motor or sensory fusion, aiming to avoid diplopia. However, as there was no visual disparity present in the optokinetic stimulation, this does not offer an explanation to the eye movement response seen in this study. As the visual binocularity supressed the response, it appears likely that other neural systems, closely linked to that of vertical vergence, may play a pivotal role in its induction during optokinetic stimulation. Consequently, when discussing the influences of non-visual systems, the vestibular pathway emerge as a potential candidate. Vertical vergence has been described as a purely vestibular reaction, conveyed through the vestibular nuclei in conjuncture with an ocular counter-roll where the eyes torque in the opposite direction of a head roll^13,22^. It is known that the vestibular nucleus is affected by visual motion, and exhibit different response patterns depending on stimuli acceleration^23^. Similarly, we know that vertical vergence adapts to visual accelerations while being generally unaffected by visual clutter, i.e. information density, while the inverse is true for the torsional response^3,6^. As such, one could suggest that the vertical vergence seen to visual rotations may be a reflexive manifestation of a visually induced activation of the vestibular nuclei. This proposed mechanism offers a possible explanation for the findings of the present study. Visual input generally decreases vestibular signalling, allowing for external fixation points to counteract the effect of self-motion on the vestibulo-ocular reflex^24^. Due to the inhibiting effect of binocularity on the divergence response, one must assume that a non-visual system acts as the primary response drive, of which the vestibular system is a prime candidate.

From an alternative perspective, vertical vergence has been suggested to have an even deeper connection to visual cues related to gravitational and vestibular information: The dorsal light reflex aims to provide visual cues for postural control, indicating the position of the sun, and have been shown to potentiate vestibular signals in early vertebrates, indicating a significant visuovestibular interaction^25^. A human correlate to this reflex have been suggested in patients with dissociated vertical vergence (DVD), a condition in which the eyes are pathologically skewed during monocular occlusion^26,27^. Theoretically, this could manifest as visually induced vertical vergence brought on by varying levels of luminosity reaching each eye. A study in healthy humans has also shown that an asymmetrical vertical phoria could be seen during a reading task under monocular conditions, the size of which depended on which eye was occluded, though the finding was not put in context with that of a dorsal light response^28^. Conversely, another study has shown that when comparing vertical vergence during dark and illuminated monocular conditions, there were no significant differences between skewing amplitudes, which has been interpreted as evidence for there being no dorsal light reflex in humans^29^. This conclusion has come into question, with the methodology of using dark and illuminated occluders not representing any physiological stimuli, instead inducing optical blur with no natural form or contours being visible^30^. While this study implemented a methodology allowing for clear visual acuity, with a rotating stimulus that corresponds to a postural shift, i.e. head roll, the differences seen between monocular and binocular viewing conditions are more likely due to a lack of fusional drive over differences in luminosity. A similar experimental setup could however be of value in discerning the nature of vertical vergence as being light-sensitive, if binocularity can be maintained while altering the relative luminosity between the eyes.

In conclusion, vertical vergence produced by optokinetic rotations is supressed by binocular vision, indicating a non-visual drive for this reflexive response. The differences between the vertical vergence and torsional response patterns further suggests that the two eye movements have separate neural pathways. We propose that vertical vergence may reflect a reflexive motor output caused by a visual activation of the vestibular nuclei. Further investigation into the area may reveal promising biomarkers for disorders affecting binocular vision and visual motion processing.

## Data Availability

Data is available upon reasonable request from the authors.
